# Aggregation, Transmission, and Toxicity of the Microtubule-Associated Protein Tau: A Complex Comprehension

**DOI:** 10.3390/ijms241915023

**Published:** 2023-10-09

**Authors:** Jiaxin Hu, Wenchi Sha, Shuangshuang Yuan, Jiarui Wu, Yunpeng Huang

**Affiliations:** 1 Key Laboratory of Systems Health Science of Zhejiang Province, School of Life Science, Hangzhou Institute for Advanced Study, University of Chinese Academy of Sciences, Hangzhou 310024, China; hujiaxin21@mails.ucas.ac.cn (J.H.); 3018001612@tju.edu.cn (W.S.); shuangshuangyuan@163.com (S.Y.); 2 Key Laboratory of Systems Biology, Hangzhou Institute for Advanced Study, University of Chinese Academy of Sciences, Chinese Academy of Sciences, Hangzhou 310024, China

**Keywords:** tau aggregates, tau toxicity, tau transmission, tauopathy

## Abstract

The microtubule-associated protein tau is an intrinsically disordered protein containing a few short and transient secondary structures. Tau physiologically associates with microtubules (MTs) for its stabilization and detaches from MTs to regulate its dynamics. Under pathological conditions, tau is abnormally modified, detaches from MTs, and forms protein aggregates in neuronal and glial cells. Tau protein aggregates can be found in a number of devastating neurodegenerative diseases known as “tauopathies”, such as Alzheimer’s disease (AD), frontotemporal dementia (FTD), corticobasal degeneration (CBD), etc. However, it is still unclear how the tau protein is compacted into ordered protein aggregates, and the toxicity of the aggregates is still debated. Fortunately, there has been considerable progress in the study of tau in recent years, particularly in the understanding of the intercellular transmission of pathological tau species, the structure of tau aggregates, and the conformational change events in the tau polymerization process. In this review, we summarize the concepts of tau protein aggregation and discuss the views on tau protein transmission and toxicity.

## 1. Introduction

The microtubule-associated protein tau (MAPT; here, tau is used for short), is one of the microtubule-associated proteins (MAPs), and plays a crucial role in regulating the dynamics of microtubules (MTs) through binding and detaching from them. Tau plays a crucial role in axonal transport [1], synaptic transmission, and connectivity [2,3,4], as well as in maintaining genomic stability and regulating gene expression [5,6,7]. However, in various neurodegenerative diseases, including Alzheimer’s disease (AD), frontotemporal dementia (FTD) with parkinsonism linked to chromosome 17 (FTDP-17), progressive supranuclear palsy (PSP), corticobasal degeneration (CBD), Pick’s disease (PiD), etc., tau is abnormally phosphorylated, leading to the formation of protein inclusions such as neurofibrillary tangles (NFTs), which are collectively referred to as “tauopathies” (Table 1) [8]. Tau is an intrinsically disordered protein lacking a defined structure, and poses challenges in understanding its mechanism of polymerization into oligomers and ordered aggregates, such as paired helical and straight filaments (PHFs and SFs), which ultimately lead to detrimental consequences [9].

**Figure 1:**
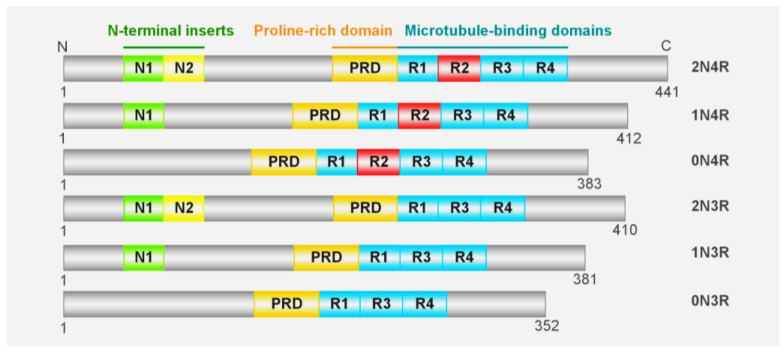
Tau isoforms. Six isoforms of microtubule-associated protein tau (MAPT) are generated through alternative splicing. In the human brain, splicing of exons 2 and 3 results in three isoforms with 2, 1, or no N-terminus insertion of 29 amino acids (referred to as 2N, 1N, and 0N). Each isoform contains a microtubule binding domain encoded by exon 10 that consists of either three repeats (3R) or four repeats (4R), resulting in the genesis of six isoforms (2N4R, 1N4R, 0N4R, 2N3R, 1N3R, and 0N3R).

Tau is encoded by the MAPT gene on chromosome 17q21.31, which contains 16 exons that can generate six isoforms due to the alternative splicing of exons 2, 3, and 10 (Figure 1). The longest isoform of tau consists of 441 residues and can be divided into the N-terminus, C-terminus, a proline-rich region, a microtubule binding region (MTBR) encompassing R1-R4 segments, and two acidic N-terminal inserts that contain 29 amino acids each [22]. The concept that tau is involved in neurodegenerative diseases is supported by the evidence that tau aggregations and mutations correlate with several neurodegenerative diseases, including AD, FTDP17, PSP, etc. Furthermore, the presence of mutations on tau is adequate to elicit neurological pathology in both human and transgenic animal models [23,24]. It is interesting to note that, similar to the propagation of tau pathology in patients, the results from animal models also provide strong evidence to support the transmissibility of tau [25,26,27]. However, the underlying mechanism of tau aggregation and toxicity, as well as its intercellular transmission, remains to be elucidated.

## 2. Tau Aggregates

**Figure 2:**
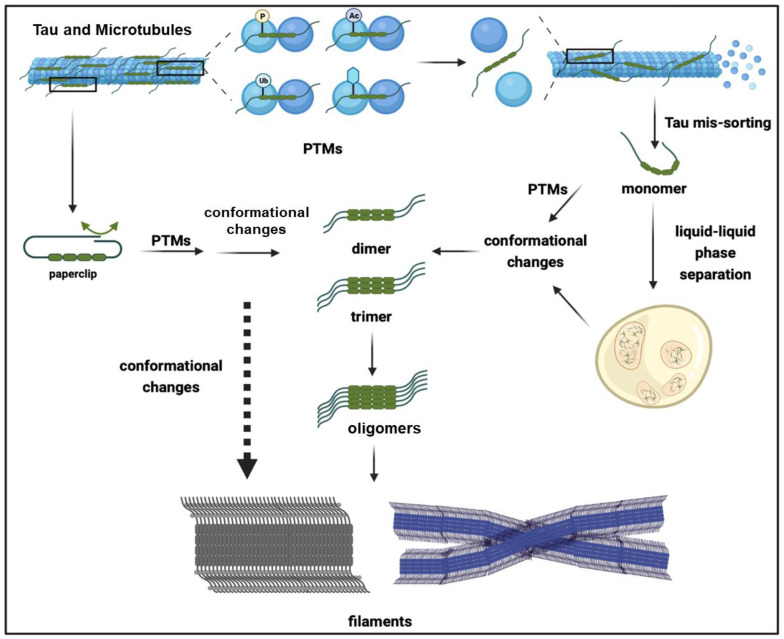
A stepwise model elucidating the process of tau aggregation. The microtubule-associated protein tau is physiologically associated with the tubulin heterodimer to maintain the stability of microtubules. Following post-translational modifications (PTMs) such as phosphorylation, tau undergoes dissociation from the tubulin heterodimer. The dissociation of tau monomers and the naturally unfolded “paper clip” tau species undergo a series of mis-sorting processes, resulting in the dendritic and somatic mislocalization of tau. This further facilitates PTMs and liquid–liquid phase separation (LLPS). The presence of PTMs and LLPS further facilitates the conformational changes in Tau, thereby exposing its aggregation-prone motifs. The intermolecular interface of tau facilitates the nucleation and formation of tau dimers and trimers, which serve as seeds to promote gradual polymerization through the extension of aggregates by the addition of tau monomers, ultimately resulting in the formation of oligomers and filaments that accompany conformational changes.

In vitro and in vivo studies have shown that the conformational change and subsequent exposure of aggregation-prone motifs serve as crucial intermediate events in the formation of oligomeric and fibrillar tau aggregates [28,29,30]. The monomeric form of tau is intrinsically unfolded, lacks a defined tertiary structure, and consists of a large proportion of random coli regions; the absence of ordered secondary and tertiary structures poses challenges in determining its structures [31]. It was proposed that the tau monomer is compacted into a “paper clip” conformation (Figure 2), in which the C-terminus folds back and interacts with the MTBR, while the N-terminus folds back over the C-terminus, forming a local compacted structure by encircling the core aggregation-prone motifs. The core aggregation-prone motifs encompass the segments “^275^VQIINK^280^” and “^306^VQRVYK^311^” on R2 and R3, respectively, which adopt a β-strand conformation [31,32,33,34], and can further facilitate polymerization through intermolecular interactions. However, intramolecular compaction prevents the intermolecular interaction of aggregation-prone motifs, thereby blocking tau aggregation [35]. Given the significant influence of intermolecular interactions on tau, factors such as heparin, PTMs, and RNA, which are capable of neutralizing and modulating the electrical properties of tau, can facilitate conformational changes and the subsequent exposure of aggregation-prone motifs.

In addition to the “paper clip” model, an intermolecular interaction can also occur through alternative mechanisms. The electricity of the tau protein is distinct across its four segments; the N-terminus of the tau protein, particularly the N1 and N2 regions, exhibits a negative charge with a combined charge of −11.8 when coupled with the proline-rich region. In contrast, the MTBR carries a positive charge of +4.5. Thereby, the N-terminus of tau can interact with the MTBR and mask its core aggregation motifs. A further neutralization of the positive charge of tau via PTMs and heparin can also hinder the intramolecular interactions, leading to the exposure of the aggregation-prone motifs [36]. Interestingly, tau monomers are predominantly present in normal brains, whereas AD brains contain a substantial proportion of tau oligomers, encompassing dimers, trimers, and high-molecular-weight polymers [37]. Accordingly, tau oligomers can be classified into two types, sarcosyl-soluble oligomers that are undetectable via AFM, and AFM-detectable oligomers, which mainly consist of sarcosyl-insoluble granular oligomers containing more than 40 mers [38,39]. In contrast to tau filaments, the structural determination of tau oligomers poses a significant challenge. The results obtained from MT-associated tau dimers indicate that they are formed through an intermolecular interaction between the N-terminus and the proline-rich region in an antiparallel manner, facilitated by electrostatic complementation involving salt bridges. Subsequently, the N-terminus, C-terminus, and MTBR regions undergo an extension to facilitate the binding of tau towards MTs. The artificial condensation of dimeric tau has been shown to increase its binding affinity towards MTs [40], particularly with respect to tubulin heterodimers [41].

The tau dimers and trimers formed under physiological conditions exhibit distinct characteristics from pathological tau oligomers, particularly in terms of their association with different phosphorylation patterns and their status. These findings suggest that normal and pathological tau oligomers possess different properties and undergo distinct biological processing [42]. Prior to the formation of oligomers, monomeric tau undergoes a series of conformational changes, particularly adopting the β-strand structure. The disulfide-cross-linked intermolecular interaction further facilitates the formation of dimers, and is accompanied by the disulfide-bond-independent intermolecular bridging of the MTBR. These dimers can further polymerize into oligomers with a distinct fate compared to the dimers that are formed physiologically [36,43].

Dimerization is likely to serve as a rate-limiting step in the aggregation of the tau protein. The tau dimer acts as the core, and it can be further expanded by the addition of monomers [44], which undergo polymerization to form multimers, and have been proven in vitro [45,46]. Moreover, tau trimers can also serve as the aggregation seed, implying that both dimers and trimers possess the potential to function as fundamental units for aggregation [37]. In particular, they are abundant in AD brains [47].

Accordingly, the formation of tau granules precedes the formation of tau filaments, which can be detected in the early stages of AD, even in the absence of AD-related symptoms. In particular, tau granules appear at Braak stages 0 and I, which implies that their genesis is an early event and may relate to the progression of AD [39]. In contrast, tau filaments can be detected at stage V instead of stages 0, I, and III, which does not exhibit a strong correlation with the early cognitive changes [48].

Unlike unstructured monomers, tau dimers, trimers, and oligomers tend to gradually adopt the β-sheet conformation. Accordingly, the content of the β-sheet conformation is increased stepwise from soluble oligomers to granular oligomers and filaments, indicating that the structure change is accompanied by tau aggregation and may facilitate the formation of higher-molecular-weight aggregates [38]. Interestingly, granular oligomers contain more β-sheet structures than randomly coli-enriched monomers, and the proportion of β-sheet structures is continuously increased in the filaments [48]. The gradual increase in β-sheet conformation is likely critical for tau polymerization [38].

Although the toxicity of tau filaments is still uncertain and debated, their structures have been investigated by several Cryo-EM studies, since they tend to form defined structures in vitro and in vivo. Unexpectedly, the structures of tau filaments are diverse in different types of tauopathy diseases [49].

On the basis of the number of protofilaments, the CBD filaments can be divided into types 1 and 2, which correspond to narrow and wide tau filaments, respectively. The ratio of type 1 to type 2 filaments in CBD patients varies from 1:1 to 1:3 [50]. The narrow tau filament consists of a single protofilament exhibiting a four-layer fold, while the wide filament comprises pairs of identical narrow filaments [50]. The CBD protofilaments’ core sequence comprises residues K274-E380 and features 11 β-strands linked by turns and arcs, forming a four-layer structure. The antiparallel stacking of the ^343^KLDFKDR^349^ motifs from two protofilaments further shapes the type 2 filaments [50]. In contrast to CBD filaments, the PHFs and SFs observed in AD patients comprise two identical protofilaments encompassing residues 306–378, with the disordered N-terminus and C-terminus forming a fuzzy coat. The filament’s core comprises eight β-sheet structures, including β1–3 of R3, β4–7 of R4, and β8 at the C-terminus. It adopts a C-shaped architecture without R1 and R2, where three β-sheets are arranged in a triangular manner. Additionally, it exhibits two regions with a cross-β architecture, wherein pairs of antiparallel β-sheets are packed together. The cross-β interface is formed by the interaction between β1–2 and β8, which is facilitated by ^306^VQIVYK^311^ and residues 373–378 from the opposing β8 sheet. The antiparallel stacking of residues ^332^PGGGQ^336^ between two helically symmetric protofilaments further contributes to the formation of PHF aggregates. In contrast, the SF stacking involves the asymmetric packing of ^321^KCGS^324^ from protofilament 1 and ^313^VDLSK^317^ from protofilament 2 at their interfaces along the helical axis [49]. 

In patients with PSP, tau polymerizes to form subcortical neurofibrillary tangles and neuropil threads, along with tufted astrocytes and oligodendroglial coiled bodies. Intriguingly, a recent study has revealed that tau exhibits the novel three-layered folded filaments in PSP, which is herein referred to as the PSP fold [51]. PSP filaments are composed of a single protofilament featuring an ordered core spanning residues 272–381 and adopting the three-layer PSP fold. This fold involves the stacking of R2–R4 and a turn at the “PGGG” motifs located at the end of each repeat. The central layer is formed by R3, with R2 and R4 packing on either side of it. Additionally, the chain undergoes another hairpin turn at the “PGGG” motif within R4, while the C-terminus forms a short fourth layer that covers the end of R2 [51]. 

Pick’s disease is classified as a 3R tauopathy. It results in the degeneration of the frontotemporal lobar, which is accompanied by the accumulation of abundant Pick bodies composed of narrow and wide 3R tau filaments. The narrow Pick’s filament consists of a single elongated protofilament, which exhibits structure differences from the PHFs observed in AD. Furthermore, the association of two narrow Pick’s protofilaments results in the formation of wide filaments. The Pick’s filament fold comprises a core consisting of residues Lys254-Phe378, which adopts nine β-strands (β1–β9) organized into four cross-β packing stacks that are connected by turns and arcs. Specifically, β1 and 2 of R1, β3–β5 of R3, and β6–β8 of R4 are tightly packed together to form a hairpin-like structure [52]. As revealed by the cryo-EM analysis, while R3, R4, and an additional 10–13 residues from the C-terminus of tau contribute to the assembly of the common folded core in tau filaments, distinct types of tau folds are observed in different tauopathy diseases, which can be further classified into three classes and eight subclasses (Table 2, see review [51]). Disease-associated folding specificity helps to elucidate the underlying mechanisms behind the diverse protein inclusions, subcellular distributions, and cell-type-specific aggregations of tau in various diseases. In particular, it helps to explain why the tau protein can deposit diversely in neuronal cell bodies, synapses, and dendritic compartments, leading to distinct pathological manifestations [53]. Interestingly, a recently discovered isoform of tau, known as w-tau, exhibits the intriguing ability to mitigate tau aggregation in AD brains [54]. Moreover, it has been demonstrated that w-tau possesses the capacity to attenuate the aggregation propensity of tau [55]. Interestingly, w-tau is generated by intron retention, which results in the absence of exon 13 and the addition of the translated intron 12 sequence after exon 12 [54].

In addition to a structural investigation, despite its inherent challenges, tau aggregation can be investigated by using several methodologies and models (Supplementary Table S1). 

## 3. Factors Facilitating Tau Aggregation 

The properties and electrostatics of tau can be influenced by various factors, including post-translational modifications (PTMs) of proteins, polyanions such as RNAs, heavy metals, heat shock proteins, membrane binding, and local condensation of tau (LLPS). PTMs such as phosphorylation, acetylation, truncation, nitration, glycation, glycosylation, and ubiquitination serve as crucial regulators of tau aggregation and toxicity. The primary impact of PTMs is to modify the local charge distribution of tau proteins. For instance, the attachment of a single phosphate group (Pi) to tau can result in an increase in the net charge by −1 on the tau protein; therefore, the enrichment of phosphor sites and phosphor groups can significantly diminish the positive charge of tau [58,59,60].

## 4. Post-Translational Modifications

The binding of phosphor groups to the MTBR can neutralize its positive charge, thereby inducing its dissociation from the MTs [61]. For instance, the binding affinity of MTs is reduced by the phosphorylation of the p-epitopes within the MTBR, such as S262/S214, and the epitopes located in the proline-rich domain, like T231 [62]. 

**Figure 3:**
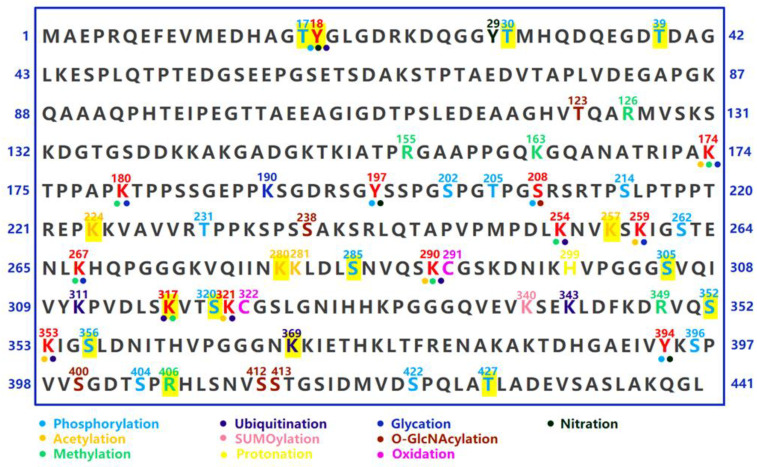
Post-translational modifications (PTMs) occur on the tau protein. The amino acid sequence of the longest tau isoform, 2N4R, is presented here. Distinctive colored dots are utilized to represent various PTMs, while the amino acids associated with tau pathology are highlighted in yellow.

Once dissociated from MTs, phosphorylated tau can further undergo pre-aggregation events. Since the charge change, the interaction between the negatively charged C-terminus and MTBR is impeded, leading to the release of the “paper clip” structure and resulting in an enhanced hydrophilic property. The subsequent intermolecular interaction of two tau proteins with the exposed aggregation-prone motifs further facilitates the formation of tau dimers and multimers [63]. Notably, some in vitro evidence suggests that the phosphorylation of the C-terminal epitopes, including Ser396, Ser404, and Ser422, does not alter the binding affinity of MTs; however, the propensity for conformational changes and aggregation is enhanced [60,64,65]. Interestingly, phosphorylation at Ser396 appears to attenuate the binding affinity of MTs in cell culture models [66,67], suggesting potential variations in tau properties across different environments. In addition to reducing the binding affinity of MTs, phosphorylation at AD-related serine (S) and threonine (T) epitopes can increase the propensity for aggregation (Figure 3). The in vitro phosphorylation of tau at AT8, AT100, AT180, and PHF-1 epitopes leads to the self-aggregation of tau, resulting in the spontaneous formation of small amorphous aggregates without the need for aggregation inducers [68]. Moreover, in vitro phosphorylation at S208 through a phosphoryl-mimetic mutation, S202E/T205E/S208E, induces Tau self-aggregation independently of an aggregation seed [69], further suggesting that phosphorylation can modulate Tau properties and drive its aggregation. The linking of polyphosphate chains also induces a conformation change in Tau, facilitating the formation of intermolecular cross-linking and aggregation [70].

Lysine (K) acetylation, achieved by introducing an acyl group to the -NH side chain, possesses the capacity to neutralize a positive charge and increase the negative charge near the lysine residue, which can also change the properties of tau [71,72]. In particular, acetylation at the K280 residue of the MTBR reduces the binding affinity of MTs (Figure 3), increases the proportion of the β-sheet structure, and enhances the self-assembly capability of tau, which can be attributed to the interaction between the hydrophobic chemical groups within the PHF6* motifs [72,73]. 

Similarly, acetylation at K281 also hampers the capacity for MT binding and enhances the propensity for aggregation [74]. Acetylation at K259, K290, K321, and K353 outside the aggregation-prone motif (PHF6* and PHF6), exhibits a similar impact on reducing the binding affinity of MTs. Surprisingly, mimicking acetylation at K321 and K353 exerts an opposing effect on tau aggregation by influencing the stability of its β-sheet structure [75]. These findings suggest that PTMs with different effects on tau conformation may differentially modulate tau aggregation [64]. 

In addition to acetylation, lysine residues on tau can undergo modifications such as ubiquitination, SUMOylation, methylation, and glycation et al., suggesting the potential for competition and crosstalk among different PTMs (Figure 3) [60,76]. 

The ubiquitination of tau exerts diverse effects. It can facilitate the degradation of tau through the ubiquitin–proteasome and autolysosome systems, while also promoting the aggregation and insoluble inclusions of the tau protein [77,78]. The ubiquitination of K18 at the N-terminus of tau disrupts aggregate formation and sequesters tau oligomers into the proteasome for degradation [79]. The ubiquitination of K63 promotes the autolysosome-mediated degradation of tau. Moreover, the ubiquitination of K48 and K63 also promotes tau aggregation due to the interaction of the enriched ubiquitin chains [79,80]. In fact, ubiquitin moieties have been detected on human-brain-derived tau aggregates, including ubiquitin chain-linked K321, K343, K353, and K369 in CBD, as well as K317/K321 in AD [81]. 

In particular, CHIP is a predominant ubiquitin ligase for tau. The deletion of CHIP results in an accelerated accumulation of non-aggregated and ubiquitin-negative forms of tau [82]. The up-regulation of CHIP expression in Cos7 cells results in the formation of ubiquitin-positive tau aggregates, which can be attenuated by Hsp70 [83]. The AD brain-derived tau conjugated to K63 ubiquitin chains is likely to exhibit a propensity for aggregation and propagation [84]. The above evidence suggests that ubiquitin conjugation not only influences the degradation of tau, but also modulates its aggregation propensity, since ubiquitin chains can provide additional interactions among tau molecules [81]. 

Although the methylation of Tau has been detected in both normal and AD human brains [85,86], the functional implications of this epigenetic modification remain to be elucidated. The methylation of both mono- and di-methylated lysine residues occurs along the entire tau protein. However, it does not impact MT polymerization unless the lysine residues of tau are methylated to a high stoichiometry. Surprisingly, even when the methylation levels are elevated, tau still maintains a disordered conformation. Additionally, overall, methylation exerts a primary influence on tau by suppressing its aggregation propensity through impairing the nucleation rate, thereby slowing down the extension of aggregates, and ultimately reducing the stability of tau filaments [86].

Intriguingly, the methylation of certain residues, such as K317, elicits an opposing effect. K317 methylation decreases tau solubility and facilitates intermolecular interactions and dimerization, which is concomitant with MT destruction [87]. In addition to lysine residues, methylation can also occur on certain arginine residues (R), including R126, R155, and R349 [88,89]. However, the functional implications of arginine methylation remain to be elucidated. Similar to acetylation, the methylation of lysine residues can induce charge neutralization and conformational changes [58], thereby influencing both intra- and intermolecular interactions [87]. The additional consequence of lysine methylation is the perturbation of other PTMs. For instance, methylation at K254 blocks Tau ubiquitination and degradation [89], while methylation at K267 influences the phosphorylation of Ser262, albeit the underlying mechanism remains unknown [90].

## 5. Heavy Metal Elements 

The disruption of heavy metal homeostasis is implicated in the pathogenesis of several tau-related neurodegenerative diseases, such as AD, which accompanies the accumulation of copper, iron, and zinc. Furthermore, the exposure to certain heavy metals also augments the incidence of AD and other tauopathies [91]. In particular, heavy metals are postulated to act as cofactors to induce tau aggregation through direct binding [92,93,94]. They possess the capability to induce conformational changes and promote phase separation of tau, thereby enhancing its propensity for aggregation [95,96]. However, it is unlikely that they share identical binding sites and kinetics.

The imbalance and accumulation of copper ions have been demonstrated in AD brains [97]. In vitro studies suggest that copper interacts with the R2 and R3 regions of tau, and that the histidine residues within tau play a vital role in the binding of copper ions. Consequently, copper induces a conformational shift in the aggregation-prone motifs, leading to an increased proportion of alpha-helix and β-sheet structures [96,98,99], facilitating tau self-assembly [98]. In addition to direct binding, copper can also catalyze the formation of intermolecular disulfide bonds, thereby promoting tau dimerization [96].

The zinc ion is abundantly accumulated in the AD brain and possesses the capacity to induce the formation of the β-sheet conformation, thus facilitating the formation of tau aggregates. Under reducing conditions, the interaction between tau and zinc is mediated by Cys291 and Cys322, with the additional involvement of certain histidine residues. Interestingly, distinct effects on tau aggregation are observed at low and high levels of zinc. In particular, low zinc levels promote the fibrillization of Tau by promoting the formation of a β-sheet structure, whereas high zinc levels induce the formation of granular aggregation. This phenomenon can be attributed to a binding site switch that occurs with increased zinc concentrations [95,100,101].

In addition, the presence of iron (Fe^2+^) facilitates the aggregation of tau by inducing a reversible conformational change through an interaction with the threonine residues [102]. Interestingly, lithium, an alkali metal, exhibits the potential to mitigate brain iron accumulation induced by tau and consequently may reduce iron-induced tau toxicity [103].

Besides the direct interaction effects, heavy metal elements can modulate the conformation and aggregation of tau by influencing the flux of PTMs; for instance, zinc and copper can impact the activities of tau kinase and phosphatase, thereby augmenting tau phosphorylation and inclusion [93]. In addition to iron accumulation correction, lithium has the ability to attenuate tau phosphorylation by inhibiting GSK3 activity and modulating the AKT and PKA levels [104,105,106,107]. Interestingly, it also directly reduces tau expression; however, the underlying mechanism remains to be elucidated [103].

## 6. Phase Separation

In the protein crowding niche, tau can be concentrated by LLPS, which has been proposed to facilitate conformational change [68,108]. Interestingly, even at a physiological concentration of as low as 1–3 µM, tau exhibits the ability to undergo LLPS. In particular, phosphorylation is crucial for tau LLPS, and unphosphorylated tau is unable to undergo LLPS, even in a crowding environment [108]. Since the phosphorylation of tau can increase its negative charge, it induces conformational changes and enhances intermolecular interactions, thereby facilitating tau LLPS. Overall, the phosphorylation of tau and site-specific phosphorylation, such as phosphorylation at the AT180 and AT8 epitopes, both enhance the propensity for LLPS [109]. In particular, at high protein concentrations exceeding 50 µM, the phosphorylated tau can undergo LLPS spontaneously, even in the absence of crowding agents. In contrast to phosphorylation, the implications of acetylation on Tau LLPS remain a subject of ongoing debate and necessitate meticulous clarification [110]. 

In addition, tau LLPS can be further facilitated by heparin, RNA, and genetic mutations of tau. In particular, even in the absence of crowding circumstances, tau can undergo spontaneous LLPS when carrying pathological mutations such as P301L and delaK280, which have been implicated in inducing β-sheet conformational changes in tau [108,111]. 

Droplets of Tau LLPS exhibit a dynamic exchange with the surrounding environment and undergo rapid gel-like condensation, which can further progress to β-sheet-enriched protein aggregates. These findings suggest that LLPS enhances aggregation propensity [108,111]. From this perspective, it is plausible that after mis-sorting from axonal MTs, the subsequent PTMs, such as phosphorylation, are able to promote the LLPS of tau, leading to its condensation (Figure 2). It has been demonstrated that LLPS can enrich the tau concentration by up to ~100-fold [111], thereby facilitating conformational changes including the extension of the N- and C-terminus, exposing the MTBR, and promoting intermolecular contact for the cluster formation of tau molecules, ultimately leading to tau aggregation and fibrillization [111,112]. Surprisingly, although the MTBR is prone to form aggregation, LLPS is predominantly reliant on the N-terminus of tau. Tau clustering in LLPS likely occurs through the interface of its N-terminus, and the removal of the N-terminus impedes LLPS, indicating that the mechanisms of tau LLPS differ from those driving tau polymerization, although LLPS can promote the aggregation of tau [111].

## 7. RNAs and RNA-Binding Proteins

Although the precise roles of RNA and RNA-binding proteins in tau aggregation remain elusive, their association with pathological tau aggregates has been demonstrated in vivo [113,114]. Tau interacts with RNA molecules through electrostatic interactions. The negatively charged phosphate backbones of RNAs bind to the positively charged segments of tau, facilitating tau condensation, particularly the LLPS [115], increasing its propensity for aggregation [114,116,117].

Structural studies of tau fibers induced by RNA molecules-reveal the incorporation of two molecular weight RNAs. The intermolecular interaction between two parallel protofilaments, mediated by the Glu391-Ala426 segments, further gives rise to an aggregation core comprising five β-strand structures [118]. The presence of RNA molecules not only serves as a structural factor that stabilizes tau aggregates, but also acts as a catalyst for tau aggregation by catalyzing the formation of tau filaments. Surprisingly, the removal of RNA molecules can lead to the unexpected dissociation of tau aggregates, indicating their crucial role as molecular glue [118,119]. In vitro evidence also suggests that tau has the ability to sequester RNA molecules and subsequently undergo co-polymerization, leading to the formation of amyloid-like fibrils [120]. 

However, the properties of tau aggregates induced by RNAs exhibit distinct dissimilarities compared to those induced by heparin. In addition to poly (U), (A), (C), and (G), tRNA, m6A-RNA, snRNAs, and snoRNAs also exhibit the capability of interacting with tau, leading to the LLPS and subsequent polymerization with distinct stoichiometry [119,120,121]. Tau LLPS is also implicated in the indirect interaction between tau and RNAs mediated by RNA-binding proteins such as TIA and HNRNPA2B1 [122,123].

## 8. Interplay between Tau Protein and Membrane Architecture (Membrane Binding)

In the AD brain, tau has been demonstrated to exhibit an association with the cellular membrane [124]. Furthermore, PHF filaments co-deposit with membrane lipids, including phosphatidylcholine (PC), cholesterol, and sphingolipid [125], thereby suggesting a link between tau pathology and its interaction with membrane lipids. Furthermore, in vivo and in vitro studies have demonstrated that tau exhibits a propensity to directly associate with membrane lipid species, which is driven by electrostatic interactions. In particular, the hydrophobic hexapeptide motifs PHF6*/PHF6 possess the ability to interact with lipids, leading to the formation of the core structure within the tau-lipid complex, which can further enhance the formation of the β-sheet structure, thereby augmenting the aggregation propensity of tau [126,127]. The binding affinity of tau and membrane lipids is predominantly determined by the intensity of the electrostatic interactions and the composition of the phospholipid headgroups [128]. The specific lipid species capable of binding to tau have been reviewed by Bok et al. [126]. Interestingly, tau has the capacity to remodel and extract phospholipids from cell membranes, leading to the formation of the tau-lipid complex. The R2 and R3 regions of tau, particularly the PHF6 motif, exhibit a robust capacity for remodeling membrane lipids. 

The formation of the tau-lipid complex facilitates a favorable shift in electrical properties [127], and the presence of a hydrophobic environment may potentially facilitate the enrichment of the tau protein, thereby promoting the intermolecular interfaces. However, it remains to be determined whether lipid binding can facilitate tau LLPS, and the role of membrane lipids in enhancing and stabilizing tau aggregates remains unclear. The binding of tau to lipids also facilitates tau propagation and transmission [126], since membrane binding enhances tau internalization and exocytosis across the cell membrane. Besides the lipids, membrane-associated proteoglycans and proteins also possess the capacity to interact with tau and contribute to tau pathology. Heparan sulphate proteoglycans (HSPGs), which are ubiquitously distributed on the extracellular matrix and plasma membrane, have the capacity to interact with tau and promote its spreading [129]. The muscarinic cholinergic receptor can interact with tau, leading to the disruption of calcium homeostasis [130]. However, the interaction between tau and lipids is predominantly limited to in vitro experiments; therefore, further investigations are warranted to elucidate the binding of tau to the membrane lipids in vivo, as well as the impact of phosphorylation and its relevance to tau aggregation.

## 9. Propagation and Transmission of Tau 

**Figure 4:**
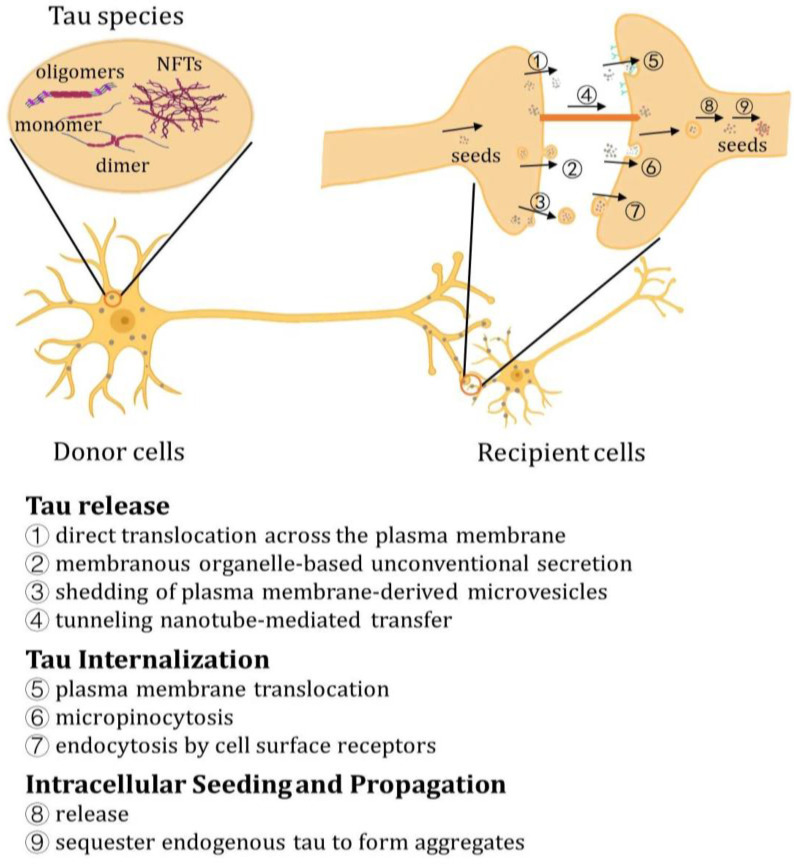
Tau transmission. The transmission of tau is initiated with the release of pathological tau species, including monomers, dimers, oligomers, and NFTs. This process is facilitated by (1) the direct translocation across the plasma membrane, (2) membranous organelle-based unconventional secretion (MOBS), (3) shedding of microvesicles derived from the plasma membrane, and (4) nanotube-mediated transfer. The extracellular tau and tau-containing vesicles can be internalized by (5) translocation across the membrane, (6) micropinocytosis, and (7) endocytosis. Subsequently, they can be (8) released into the recipient cells, (9) seeding the endogenous tau to undergo conformational change and polymerization.

Similar to prions, pathological tau can serve as a nucleating seed for aggregation, inducing the conformational transformation of intrinsically disordered monomers of tau. This process leads to the formation of a conformation that is prone to aggregation and further polymerization, ultimately resulting in the expansion of tau toxicity, which is demonstrated as “tau transmission and propagation” [131,132,133,134] (Figure 4). 

The transmission process involves the formation of pathological tau species, which serve as propagating seeds and can be secreted from donor cells into the extracellular space. Subsequently, they are internalized by recipient cells, where they act as seeds to facilitate the sorting and conversion of normal monomeric tau into aggregation-prone species [135,136]. The propagation and transmission of Tau have been unveiled and can be investigated through various models and methodologies (Supplementary Table S2).

## 10. Release of Tau

In the absence of a canonical secretion signal peptide, tau is likely secreted by the unconventional protein secretory pathway (UPS) rather than the conventional ER-Golgi secretory pathway. According to current knowledge, tau can be secreted through three mechanisms: (1) direct translocation across the plasma membrane; (2) membranous organelle-based unconventional secretion (MOBS); and (3) shedding of plasma membrane-derived microvesicles [135].

It has been demonstrated that the direct translocation of tau across the plasma membrane is facilitated by its binding to certain lipid species located on the inner leaflet of the plasma membrane, such as PI (4,5)P2. Furthermore, tau is released from the membrane, which occurs through an interaction with HSPGs that are present on the outer leaflet of the plasma membrane [135], which is similar to the release process of FGF2, and can be enhanced by phosphorylated tau [137]. In fact, elevated levels of HSPGs such as syndecan 4 and serglycin have been implicated in the presence of amyloid and tau pathology [138]. The inhibition of HSPGs and their interaction with tau has demonstrated a reduction in in vivo tau propagation [129], thus highlighting the critical role played by HSPGs in facilitating tau release. It is still not clear whether the pathological species of tau can produce a transmembrane pore similar to FGF2, and additional investigation is required [139].

Moreover, intracellular tau can be packaged within membranous organelles, such as exosomes, which then fuse with the plasma membrane, thus facilitating the release of organelle-encapsulated tau into the extracellular space. The exosome-mediated secretion of tau is initiated by the engulfment of tau into intraluminal vesicles derived from the inward budding of the late endosome. These vesicles contain hyperphosphorylated species of both monomeric and aggregated forms of tau [135,140,141]. In particular, hyperphosphorylated tau has been detected in the exosome fraction of the cerebrospinal fluid in Alzheimer’s, suggesting that tau is potentially secreted by exosomes [141], and it has been verified in cell cultures and mouse models [140,142].

Although exosome-mediated transmission only contributes to a small fraction of tau propagation, patient-derived exosomes have demonstrated the ability to propagate tau pathology in cell culture and mouse models. Moreover, inhibiting exosome formation can effectively impede tau propagation, providing evidence to suggest that exosome-mediated tau secretion is involved in this process [135,143]. In particular, exosome-mediated tau secretion also contributes to the trans-synaptic transmission of tau, a process that is dependent on synaptic connectivity and neuronal transmission activity, although the precise mechanism is not fully understood [140].

It has been suggested that the disruption of the fusion event between the autophagosome and the lysosome can potentiate autophagosome-mediated cargo release [135]. Although the underlying mechanism remains elusive, impaired autophagy and the accumulation of autophagic vacuoles have been broadly demonstrated in AD [144]. Once engulfed by autophagosomes, tau can either be secreted by the fusion of autophagosomes with the plasma membrane [145], or degraded by the fusion of autophagosomes with lysosomes [135]. Tau is able to block autophagosome–lysosome fusion by downregulating the expression of the IST1 factor associated with ESCRT-III (IST1). Given that the interaction between IST1 and charged multivesicular body protein 2B\4B (CHMP2B\CHMP4B) plays a crucial role in the assembly of the endosomal sorting complex required for transport complex III (ESCRT-III complex), tau can hinder the formation of the ESCRT-III complex, thereby inhibiting the activity of autophagosome–lysosome-mediated protein degradation. Moreover, tau can disrupt lysosome-mediated protein degradation by enlisting and sequestering FKBP4, resulting in diminished lysosome clustering [146,147]. Consequently, tau may enhance autophagosome-mediated secretion by inhibiting the functionality of the autophagosome–lysosome pathway [148]. In contrast, augmenting autophagic flux by inhibiting p300/CBP activity can conversely attenuate the secretion and propagation of tau [145,149].

Unlike exosomes, ectosomes are larger extracellular vesicles that are shed directly from the plasma membrane through budding [150]. Tau-containing ectosomes have been characterized in the cerebrospinal fluids in Alzheimer’s [151]; in particular, tau can indeed be secreted by ectosomes in cell culture and rat models [152]. However, the precise mechanism underlying ectosome-mediated tau secretion remains to be elucidated, particularly regarding its potential involvement in tau propagation in vivo.

The exocytosis of tau can also be facilitated by the secretion of late endosomes/lysosomes [153]. In particular, the up-regulation of the late endosome/lysosome-associated small GTPase Rab7 has been observed in post-mortem AD brains, suggesting a potential increase in Rab7-mediated late endosome/lysosome secretion that may contribute to disease progression, which has been verified in cell culture models. The reduction in Rab7 expression leads to a decrease in tau secretion, while the up-regulation of Rab7 expression exerts the opposite effect [154,155,156]. Furthermore, the secretion of tau through late endosome/lysosome-mediated pathways is also modulated by Hsc70 and its partner DnaJC5, which has been termed as misfolding-associated protein secretion (MAPS) [153,157,158]. The MAPS pathway is initiated by the recognition of tau by the ER-bound chaperone USP19, followed by the processing of tau by Hsc70 and DNAJ5C. This leads to the translocation of tau into the lumen of ER-associated late endosomes, which are then secreted into the extracellular space via the SNAP23- and syntaxin-4-mediated plasma membrane fusion of tau-containing late endosomes/lysosomes [153,159], leading to the free distribution of tau in the extracellular milieu, thereby facilitating its propagation [157]. The MAPS pathway is likely to function in parallel with the proteasome-mediated protein degradation system, serving as an additional mechanism for maintaining protein quality control (PQC). The initiation of the proteasome pathway is also facilitated by the USP19-mediated sorting of protein clients, and its functional disruption indeed has the potential to augment MAPS [160]. It is worth noting that the accumulation of tau in AD may impede proteasome function, potentially leading to the pathological promotion of MAPS-mediated tau secretion as a compensatory mechanism for impaired PQC, thereby reducing the intracellular levels of misfolded tau [161,162,163].

Nanotubes represent cellular protrusions that facilitate the direct intercellular delivery of cargos such as prion proteins, viruses, and organelles. F-actin-rich tunneling nanotubes are capable of facilitating the intercellular transportation of tau, thereby facilitating tau transmission [134,164]. Interestingly, nanotubes possess the capacity to facilitate the transportation of tau fibrils between neuronal cells with the aid of Actin [165,166]. In particular, the formation of nanotubes can be promoted via extracellular tau, which is likely involved in the formation of nanotubes [165]. Cellular stress, such as the accumulation of misfolded proteins like alpha-synuclein and tau, along with lysosomal and proteasomal dysfunction, may drive the formation of nanotubes to alleviate protein entrapment and aggregation-related stress [136,167]. Furthermore, the involvement of nanotubes in the propagation of tau in animal models and patients remains unclear.

## 11. Tau Internalization

The internalization of extracellular tau and tau-containing organelles is predominantly reliant on endocytosis. The internalization of tau fibrils is mediated by micropinocytosis through the invagination of the plasma membrane, which is a process modulated by Actin. In particular, the binding and internalization of tau fibrils are modulated by HSPGs on the cell surface, and inhibiting the binding of tau and HSPGs can prevent the uptake and propagation of tau [129]. In contrast to fibrils, the internalization of low-molecular-weight species such as tau oligomers is mediated via bulk endocytosis, which is akin to the fluid-phase endocytosis of dextran, and is mediated by dynamin. Relevantly, bulk endocytosis is thereby able to enrich tau within endosomes [168,169]. In particular, monomeric tau species are also able to be slowly internalized by actin-dependent micropinocytosis and rapidly internalized via dynamin-dependent endocytosis [135]. In addition, the endocytosis of free tau species, including monomeric tau and tau oligomers, is also modulated by cell surface receptors such as LRP1. The inhibition of LRP1 significantly impedes the propagation of tau in mice, suggesting that interfering with its internalization can potentially reduce tau pathology. However, it should be noted that the inhibition of LRP1 only partially reverses the uptake of sonicated Tau fibrils [129,170].

The uptake of tau exosomes is modulated via filopodia-mediated internalization, involving filopodial surfing, grabbing, and pulling motions. This dynamic filopodia extends to endocytic hot spots to facilitate the delivery of exosomes into the endosomes [171].

## 12. Intracellular Seeding and Propagation of Tau

Once internalized, tau can be released from endosomes or macropinosomes by permeabilizing through vesicular membranes, although the precise mechanism underlying this process remains to be elucidated. Internalized tau aggregates can further induce damage to the membrane of endosomes [172]. Importantly, tau has the ability to interact with vesicular membranes by sequestering phospholipids, thereby leading to membrane rupture [127]. In fact, other protein aggregates like alpha-synuclein can also cause a similar rupture of endosome membranes [173].

Following its release, tau can induce the formation and aggregation of endogenous tau by adopting a conformation that is prone to aggregation, resembling the behavior exhibited by intracellular tau seeds [36,120,132,174,175]. Remarkably, monomeric tau also has the ability to sequester endogenous tau and form the nucleation core, thus facilitating tau polymerization in a prion-like manner [36,176]. The internalized monomeric tau can also activate calpain, thereby facilitating the cleavage of endogenous tau [177]. In accordance with the propagation of tau, tau pathology exhibits gradual progression and spread patterns, which initially manifest in the transentorhinal cortex, and subsequently spread to the entorhinal cortex, hippocampus, deeper cortical layers, and subcortical nuclei [178,179,180].

## 13. Revisiting the Pathogenic Mechanisms of Tau Toxicity 

**Figure 5:**
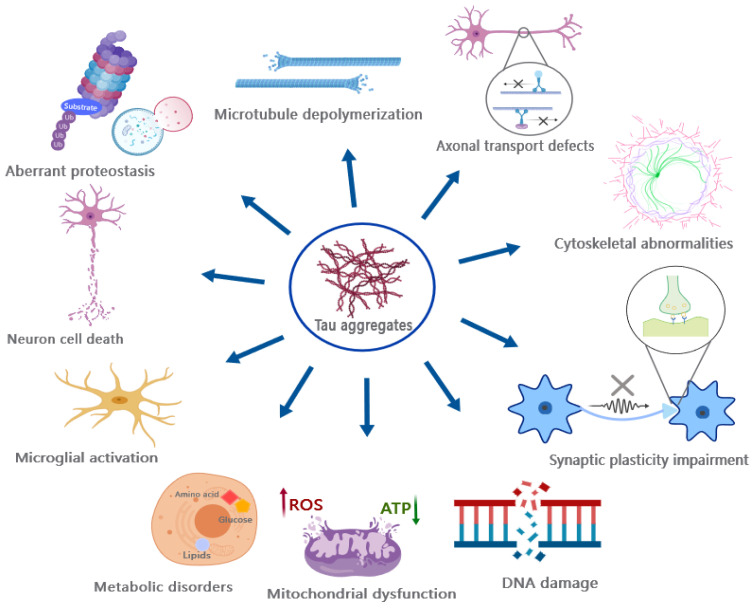
The toxic effects of tau aggregates. The deleterious effects of Tau aggregates encompass a variety of biological processes, including destabilization of microtubules and cytoskeletons, impairment of axonal transport and synaptic plasticity, disruption of mitochondrial function and proteostasis, hyperactivation of microglia, and induction of neuronal cell death, among others.

Given its pivotal role in regulating microtubule dynamics, aberrant PTMs and other factors can disrupt the normal physiological function of tau by altering its properties, subcellular localization, and promoting the formation of insoluble tau aggregates, thereby leading to at least two detrimental toxic effects (Figure 5): (1) the loss of normal physiological function and (2) the gain of toxic effects resulting from tau aggregates.

Physiologically, tau binds to MTs and stabilizes MTs by acting as a spacer between tubulin dimers [181,182]. However, hyperphosphorylation of tau abolishes its ability to bind MTs, resulting in their dissociation [183,184,185] and subsequent destabilization. Since MTs serve as the structural skeleton and corridor for efficient axonal transport, they facilitate the movement of cargo between the neuronal cell body and their axon terminals, such as nutrients, signaling molecules, and organelles [186,187]. Therefore, the dissociation of MTs leads to the disruption of axonal transport and synaptic dysfunction [188,189], manifesting as cognitive decline, memory loss, and other associated symptoms [190,191].

The toxicological effects of tau aggregates, particularly the oligomers, exhibit a complex and diverse nature (Figure 5) [192]. For instance, tau oligomers have been shown to exert detrimental effects on mitochondria, disrupt fast axonal transport, impair genomic stability, interfere with synaptic transmission and function, destabilize MTs and the cytoskeleton including the Actin network, disrupt protein degradation systems, as well as induce cell death such as apoptosis [192,193,194,195].

The toxicity of NFTs and filaments remains debated despite their association with tauopathy as one of the pathological features. However, it is important to note that neuronal dysfunction and cell death precede the formation of NFTs in both human patients and animal models, suggesting that NFTs may not be the primary causative factor responsible for exerting tau toxicity [193,196,197], although several lines of evidence suggest that there is a correlation between neuronal apoptosis and the formation of NFTs [24], and the neuronal dysfunction and behavioral changes in tauopathy animals coincide with the occurrence of NFTs [198]. A precise study by Santacruz et al. demonstrates that tau toxicity is independent of the accumulation of NFTs, which is revealed by expressing a repressible human tau variant in mice. The suppression of tau expression significantly attenuates tau toxicity in mice, while the formation of NFTs continues [199]. The assessment of the toxicity of different tau species in vivo is challenging since monomeric and oligomeric tau species also serve as precursors of NFTs. Thereby, animals with NFTs exhibit the coexistence of monomeric and oligomeric tau species, implicating that the toxic effects associated with NFTs may also be caused by the intermediate oligomeric tau and the hyperphosphorylated monomeric tau.

It is noteworthy that tau toxicity extends beyond synapses, MTs, and axons as mentioned [192]. In addition, tau can interfere with various biological processes, and induce genomic instability, neuroinflammation, metabolic disorders, and membrane remodeling [192]. Neuroinflammation has been suggested as an important consequence of pathological tau accumulation in various neurodegenerative diseases, such as FTD, Pick’s disease, and PSP, which can result in synaptic clearance and neuronal damage [200,201,202,203]. Additional evidence from mouse models has demonstrated that tau possesses the capacity to elicit hyperactivated inflammation, encompassing the excessive activation of microglia and T-cells [204,205]. Interestingly, the inhibition of microglial and T-cell activation has been shown to effectively block tau pathology [205], thereby suggesting a significant contribution of inflammatory responses in tau pathology.

A new perspective regarding tau toxicity is that it has the potential to disrupt cellular metabolism [206,207]. Tau deposition is correlated with impaired metabolisms of glucose, norepinephrine, and purine [208,209], reduced glucose utilization and oxidative phosphorylation activity [209,210], as well as the interference of the arginine, ornithine, and methionine metabolisms [211]. Although the metabolic decline in AD can be attributed to the synergistic effect of Aβ and tau [207], it is plausible that tau itself exerts a significant influence on inducing metabolic disturbances. The expression of only the tauP301L mutant in a transgenic mouse model can eventually result in metabolic disturbance [212,213], including the impaired metabolism of glucose and glutamate, which is attributed to the reduced expression of metabolic enzymes [214]. Additionally, it has been demonstrated that induced neurons (iNs) derived from Alzheimer’s fibroblasts undergo a metabolic switch to facilitate aerobic glycolysis as a result of a pathological isoform transition of pyruvate kinase M [215]. Furthermore, tau can disrupt energy biogenesis by interfering with the activity of mitochondrial complex-I [214]. The interaction between tau and mitochondrial proteins like NDUFS5, 6, NDUFA8, etc., can also impact the mitochondrial proteomes [216]. However, the diverse metabolic perturbing effects of tau, the link between metabolic disturbance and tau toxicity, and the underlying mechanisms remain poorly understood.

## 14. Discussion

The properties and structures of tau have been shown to be diverse in various tauopathy diseases, which highlights the complexity of tau polymerization. The mechanism by which tau adopts the aggregation process in vivo remains incompletely understood despite the initial electrical change and subsequent gradual conformation shift model that have been demonstrated. The diversity of tau aggregation courses may hinder the efficacy of targeted compounds and antibodies, necessitating careful consideration in future therapeutic approaches for tauopathies.

To propagate its pathology, tau undergoes a series of transmission events involving the release of transmissible seeds and subsequent internalization by recipient cells. It is noteworthy that the modulation of the tau transmission process, such as perturbing HSPGs and endocytosis, has the potential to slow down tau propagation in animal models, thereby offering a promising avenue for endeavors aimed at attenuating the progression of tau pathology in vivo.

Importantly, the multifaceted downstream consequences of tau dysfunction and aggregation necessitate a comprehensive approach, and targeting a single consequence may yield limited efficacy. It is worth noting that prioritizing the upstream driving forces and the tau protein itself holds greater potential for mitigating tau toxicity.

## Figures and Tables

**Table 1:** Relevance of microtubule-associated protein tau deposition in neurodegenerative disorders.

Tau Isoforms	Tauopathies	Neuropathological Hallmarks	References
3R Tau	Pick’s disease (PiD)	Ballooned neurons, gliosis, Pick bodies	[10,11]
3R + 4R Tau	Primary age-related tauopathy (PART)	Neurofibrillary tangles (NFTs), absence of amyloid (Aβ) plaques	[12]
	Alzheimer’s disease (AD)	NFTs, Aβ plaques, dystrophic neurites Plaques, pretangle neurons, tangle neurons	[11]
	Familial British dementia (FBD)	Cerebral amyloid angiopathy (CAA), parenchymal Aβ plaques, NFTs	[13]
	Familial Danish dementia (FDD)	CAA, NFTs, Aβ plaques, Danish amyloid	[13]
	Chronic traumatic encephalopathy (CTE)	NFTs, astrocytic tangles	[14]
4R Tau	Progressive supranuclear paralysis (PSP)	NFTs, tufted tau-positive astrocytes, coiled bodies	[15]
	Globular glial tauopathy (GGT)	Globular oligodendrocytic inclusions, globular astrocytic inclusions	[16]
	Corticobasal degeneration (CBD)	Astrocytic plaques, preganglionic neurons, coiled bodies, argyrophilic threads	[17,18]
	Argyrophilic grain disease (AGD)	Argyrophilic grains, small spindle-shaped lesions, pretangle neurons, oligodendroglial coiled bodies	[19,20]
	Aging-related tau astrogliopathy (ARTAG)	Thorny or granular/fuzzy astrocytic tau	[21]

**Table 2:** Monomeric, oligomeric, and filamentary forms of tau protein.

Tau Species	Molecular Weight (kDa)	Features	Isoforms or Compositions	Folding Types	References
Monomer	55–74	soluble	tau0N3R, tau1N3R, tau2N3R, tau0N4R, tau1N4R, and tau2N4R		[56]
Dimer	180	cysteine-dependent and reducible	2 Tau monomers		[45]
	130	cysteine-independent and unreducible			[57]
Small oligomers	300–500	soluble	6–8 Tau monomers		[43]
Granular oligomers	1800	insoluble, granule	40 Tau monomers		
Filaments		two-layered	3R	Narrow Pick filaments	[51]
				Wide Pick filaments	
		three-layered	4R	PSP fold	
				GPT fold	
				GGT type 1, type 2, and type 3 folds	
		four-layered		AGD type 1, type 2 folds	
				CBD fold	
		two-layered	3R + 4R	AD fold (including FBD and FDD folds)	
				CTE fold	

## Data Availability

Not applicable.

## Supplementary Materials

The following supporting information can be downloaded at https://www.mdpi.com/article/10.3390/ijms241915023/s1. References [217,218,219,220,221,222,223,224,225,226,227,228,229,230,231,232,233,234,235,236,237,238,239,240,241,242,243,244,245,246,247,248,249,250,251] are cited in the Supplementary Materials.

## Abbreviations

AD	Alzheimer’s disease
AFM	Atomic force microscopy
Aβ	Amyloid-beta
CBD	Corticobasal degeneration
ER	Endoplasmic reticulum
FTD	Frontotemporal dementia
FTDP-17	Frontotemporal dementia with parkinsonism linked to chromosome 17
HSPGs	Heparan sulphate proteoglycans
iNs	Induced neurons
LLPS	Liquid-liquid phase separation
LRP1	Low-density lipoprotein receptor-related protein 1
MAPS	Misfolding-associated protein secretion
MAPT	Microtubule-associated protein tau
MOBS	Membranous organelle-based unconventional secretion
MTBR	Microtubule-binding region
MTs	Microtubules
NFTs	Neurofibrillary tangles
PC	Phosphatidylcholine
PHFs	Paired helical filaments
SFs	Straight filaments
PiD	Pick’s disease
PQC	Protein quality control
PSP	Progressive supranuclear palsy
PTMs	Protein translational modifications
SFs	Straight filaments
UPS	Unconventional protein secretory
