# Supplementary material for: Aggregation, Transmission, and Toxicity of the Microtubule-Associated Protein Tau: A Complex Comprehension
Source: Int J Mol Sci. 2023 Oct 9;24(19):15023. doi: 10.3390/ijms241915023 (PMC10573976; doi:10.3390/ijms241915023)
Supplement: Supplementary file 1 [file ijms-24-15023-s001.zip › ijms-2501196-supplementary.pdf]

International Journal of Molecular Sciences

MDPI

Supplementary Table S1. In vitro and in vivo models\methods for investigating tau aggregation

	Models\methods	Summary	References
In vitro	Continuous mode thioflavin T (ThT) aggregation assay	Heparin-induced tau aggregation was subsequently monitored for aggregation kinetics in a continuous mode using ThT fluorescence.	[217]
	Fluorescent biomarkers and thioflavin staining techniques were employed	Transduction of pathogenically mutated tau using a fluorescence reporter in cell models, exhibiting positive staining for ThT and displaying markers indicative of neurotoxicity.	[218]
	Solution and solid-state NMR	Solution NMR is employed to investigate the secondary structure propensities of monomeric tau and tau aggregation intermediates, including oligomers, while solid-state NMR is utilized for studying tau filaments.	[219]
	Fluorescence resonance energy transfer (FRET) sensor	The assembly of tau protein triggers the activation of the FRET sensor, facilitating efficient energy transfer from a donor fluorophore to an acceptor fluorophore.	[220]
	BiFC turn-on and turn-off sensor	Fluorophore size was minimized by fragmenting a single fluorescence protein into two components, fluorescence activation only upon association of the tau proteins.	[220]
	ClearTau method	Generation of tau fibrils devoid of any co-factors encompassing full-length tau, utilization of seeded tau for thioflavin S (ThS) fluorescence aggregation assay.	[221]
	Human induced pluripotent stem cell (hiPSC)-derived neuronal model for tau seeding	hiPSC-derived cortical neurons are inoculated with AD brain-derived pathogenic tau species or recombinant tau fibrils.	[222]
	iPSC-derived forebrain organoids (FBOs)	FBOs are generated by modulating the concentration of FGF2 in iPSC culture medium, and subsequent AAV injection of mutant tau into FBOs leads to the formation of tau aggregates comprising tau fibrils.	[223]
	Microparticle immunocapture assay	A microparticle-based, antibody-mediated capture platform is developed for the quantification and sizing of tau aggregates, with selective exclusion of monomers.	[224]

In vivo	*Drosophila* Tau^{LUM}	Mutant tau proteins are genetically fused with two luminescent sensors, enabling longitudinal measurements of tau multimerization in a living and aging brain.	[225]
	Noninvasive in vivo imaging	Highly specific single-domain tau antibodies exhibiting enhanced brain penetration and distribution.	[226]
	Fluorescence-guided Bond-Selective Intensity Diffraction Tomography (FBS-IDT)	Three-dimensional visualization of the β-sheet structure in tau fibrils.	[227]
	Positron emission tomography (PET) and single-photon emission computed tomography (SPECT)	Radiolabeling is employed to visualize tau aggregates for imaging.	[228-230]

Supplementary Table S2. In vitro and in vivo models\methods for investigating tau propagation

	Model/Methods		Advantages	Limitations	References
In vitro models \methods	Cell culture models	Primary neuronal cultures or immortalized neuronal cell lines are exposed to tau aggregates isolated from tauopathies disease.	Facilitating the observation of internalization and intercellular propagation. Easy to culture and manipulate.	Refrain from fully capturing the intricate neuronal networks within the brain.	[145,231–233]
	Co-culture models	Neurons directly derived and cultured from animal brains are co-cultured with cells expressing tau aggregates.	Enables the visualization of tau uptake and propagation within and between cells, thereby offering a more physiologically relevant in vivo-like setting compared to conventional cell culture models.	Culturing and maintaining become more challenging, while experimental conditions are subject to less control.	[234–236]
	Brain slice	Thin slices of murine brain tissue are main-	Tau aggregates can be applied onto these	Limited to the investigation of	[237–239]

	Model/Methods		Advantages	Limitations	References
	cultures	tained ex vivo to investigate the propagation of tau.	slices, allowing for longitudinal monitoring of tau pathology.	specific brain regions, this study may not fully capture in vivo conditions and could be confounded by altered tissue physiology.	
	Microfluidic device models	In vitro models employing chambers for physical segregation of cell bodies and axons facilitate the investigation of tau transmission along axons.	These models allow for direct observation of tau propagation along axons and offer some degree of experimental control.	The experimental conditions may not fully replicate the in vivo environment and necessitate the use of specialized equipment.	[240-242]
In vivo models \methods	Injections of tau aggregates	Misfolded forms of tau protein, which are associated with neurodegenerative disorders, are administered to animal models for the purpose of comprehending disease pathogenesis and evaluating potential therapeutic interventions, thereby inducing disease-like features in the recipient animals.	Can directly induce and investigate the propagation of tau pathology	Invasive procedures may not comprehensively capture the natural progression of tau pathology.	[243-246]
	Viral vector models	Viral vectors, such as lentivirus or adeno-associated virus, are commonly utilized to facilitate the efficient delivery of human tau genes into specific brain regions or cell types for the purpose of modulating the expression of htau.	This technique enables researchers to investigate gene function within the brain and its implications in disease pathology. Precise regulation of tau expression and subcellular localization.	Confined to the examination of specific cerebral regions.	[143,247,248]

Model/Methods		Advantages	Limitations	References
Recombinant animal model expressing human tau protein	Genetically modified animals carrying and expressing the human tau gene are utilized in scientific research to elucidate the functionality of tau within the human brain and its involvement in neurodegenerative disorders.	By manipulating human tau to replicate mutations observed in these diseases, researchers can investigate disease mechanisms and evaluate potential therapeutic interventions. Enables the investigation of tau transmission in a whole organism and facilitates the modeling of progressive tau pathology over time.	May not fully recapitulate the complexity of human disease and their maintenance is both costly and time-consuming.	[249–251]
